# Comparative study of novel dosing schedules for interrupted immunotherapy for allergic rhinitis

**DOI:** 10.1002/clt2.12147

**Published:** 2022-04-15

**Authors:** Lin Xi, Chengshuo Wang, Yunbo Gao, Yuan Zhang, Luo Zhang

**Affiliations:** ^1^ Department of Allergy Beijing TongRen Hospital Capital Medical University Beijing China; ^2^ Beijing Key Laboratory of Nasal Diseases Beijing Institute of Otolaryngology Beijing China; ^3^ Department of Otolaryngology Head and Neck Surgery Beijing TongRen Hospital Capital Medical University Beijing China

To the Editor,

Allergic rhinitis (AR) management includes allergen avoidance, pharmacotherapy, allergen‐specific immunotherapy (AIT), and patient education. AIT is currently the only curative intervention that can potentially change the natural course of allergic disease.^1^ Patients receiving AIT, particularly subcutaneous immunotherapy (SCIT), are required to go to the local hospital to complete treatment. There are no evidence‐based guidelines on dose adjustments for missed SCIT doses to date, it is customary to repeat or reduce the dose when the interval between injections is prolonged,^2^ and an interruption of more than 16 weeks in the maintenance period requires that the treatment be reinstituted from the beginning.^3^ The implementing novel strategies to effectively manage AR patients receiving SCIT is critically important especially during the coronavirus disease 2019 (COVID‐19) pandemic.^4^ Here, we developed a novel dose adjustment schedule for interrupted SCIT for AR.

This was a prospective randomized open‐label single‐center clinical trial (NCT04929093) undertaken in Department of Allergy in Beijing TongRen Hospital from July 11, 2020 to April 30, 2021. 68 eligible subjects were recruited from a cohort of dust mite (DM) AR outpatients who were receiving cluster SCIT (Alutard SQ, ALK‐Abelló)^5^ and had discontinued injection for more than 16 weeks. These participants had reached the maintenance period and the overall treatment time was more than 1 year but less than 2 years and they were randomized to receive either novel (*N* = 34) or conventional (*N* = 34) dose adjustment schedules using a computer‐generated randomization code. Briefly, the conventional dose adjustment schedule restarted from the beginning (Vial 1, 10 SQ dose),^3^ whereas the novel dose adjustment schedule began with Vial 4, 10,000 SQ dose. Another cohort that received continuous cluster SCIT during the same period was included as control (*N* = 34). Clarityne (tablet) and budesonide (nasal spray) were permitted as rescue medications in all the subjects.

As a routine, all the patients receiving SCIT in our clinic scored the past week's total nasal symptom scores (TNSS) (0–12) (nasal obstruction, nasal itching, sneezing, and rhinorrhea) and medication scores (MS) (0–3) before each injection. The time points of efficacy evaluation involved in present study include the original baseline, the last injection before interruption (last injection), first re‐injection (R0), re‐reaching maintenance phase dose (Vial 4, 100,000 SQ; R3/6), and post re‐treatment 26 weeks (R26) as illustrated in Figure 1A. The primary clinical efficacy of re‐SCIT was evaluated using the daily Combined Symptom and Medication Score (CSMS) between R26 and R0.^6^ The local reactions (LRs) and systemic reactions (SRs) were recorded.^3^

**FIGURE 1:**
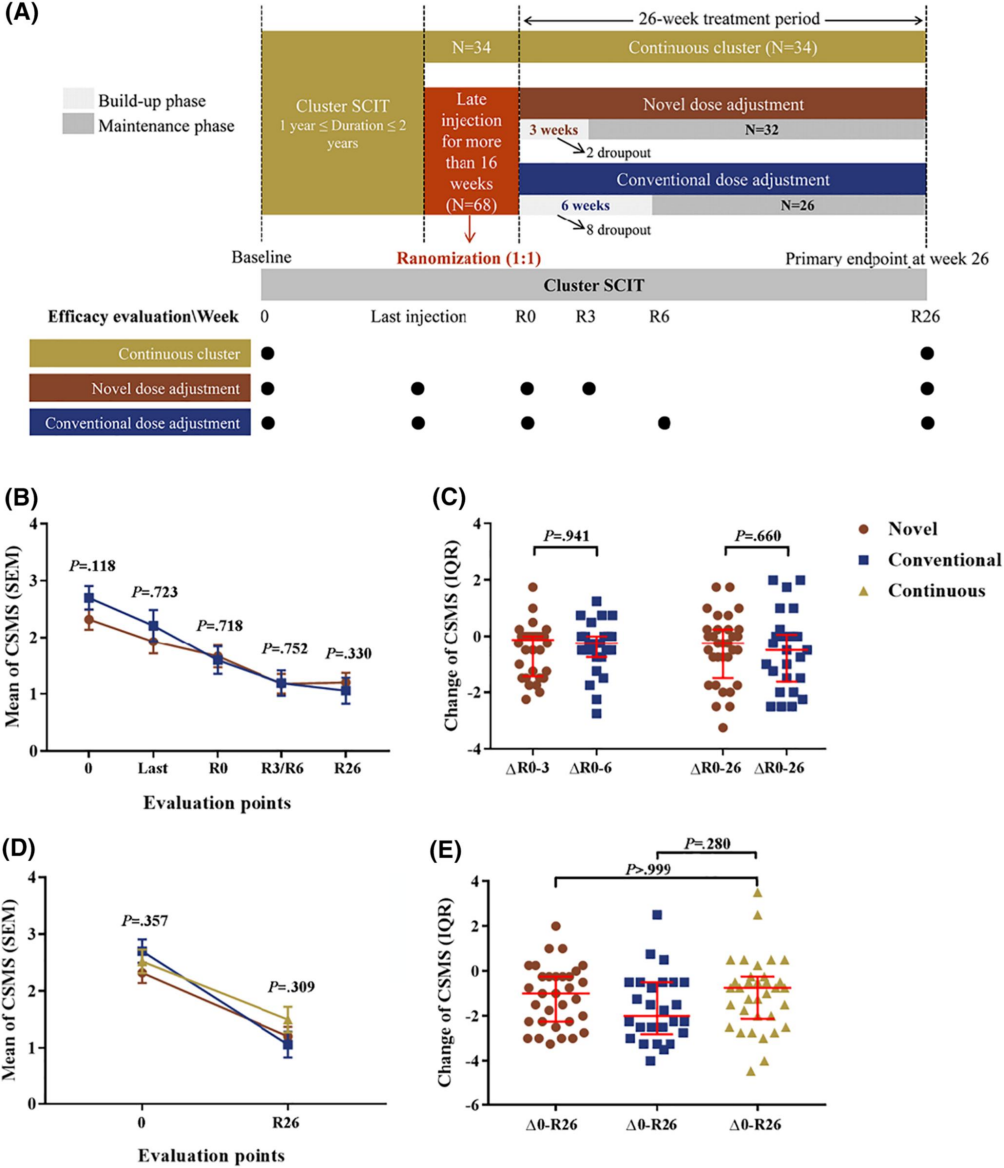
Study design and clinical efficacy among the novel and conventional dose adjustment schedules, and continuous schedule. CSMS, combined symptom and medication score; IQR, interquartile range; R, restart treatment; SCIT, subcutaneous immunotherapy; SEM, standard error of mean

The characteristics of the study population are shown in Table E1. We finally analyzed data from 26, 32, and 34 patients to evaluate efficacy in the conventional dose adjustment group, the novel dose adjustment group, and the continuous SCIT group, respectively. There were no significant differences concerning age, gender, *Der p* sIgE, total IgE, or the *Der f* combination rate among three groups at the initiation of SCIT. The baseline CSMS, TNSS, and MS among the groups were also comparable. In patients whose treatment was interrupted, the late injection time was 17.9 and 17.3 weeks in the conventional and novel dose groups respectively.

Concerning the comparison of clinical efficacy of novel and conventional dosing schedules, the CSMS at last injection and R0 between novel and conventional dosing schedules were comparable (Figure 1B). During the retreatment period, CSMS at the maintenance dose (R3/R6) as well as at R26 between novel and conventional schedule‐receiving subjects were also comparable (Figure 1B). Moreover, the changes in CSMS from R0 to R3/R6 as well as R0 to R26 in the novel and conventional groups demonstrated no significant difference (Figure 1C). Further, the similar results were obtained regarding TNSS and MS between groups.

AIT for AR is effective,^7^ but whether delayed injection for long time affects the overall therapeutic efficacy remains unclear. Here CSMS was significantly reduced by the end of R26 (SCIT for a total of about two years) compared to baseline in both continuous and interrupted schedules group (Figure 1D), and no significant differences of CSMS changes between baseline and R26 were observed in patients treated with continuous or interrupted schedule (Figure 1E). Furthermore, among all the interrupted schedule receiving subjects, there was no significant rebound regarding CSMS of R0 compared with last injection before delay (*p* = 0.074). These findings suggest that late injection may not affect the overall efficacy as long as the treatment continues.

There were 2 LRs in novel group and 7 LRs in conventional group during build‐up phase, while there were 2 LRs in novel group and 3 in conventional group in maintenance period, indicating no differences between groups in frequency of LR (*p* = 0.560). There were no SRs in both groups. Eight (23.5%) in conventional group and two (5.9%) in novel group dropped out during build‐up phase.

Limitations of this study include the small sample size that may possibly increase the risk of type II errors and especially be insufficient to determine safety adequately in the more rapid reinstituted of SCIT. Moreover, the absence of randomization for grouping and the inconsistent time intervals for evaluation among three groups may potentially introduce bias. Further prospective randomized studies involving a larger well‐characterized study populations are needed to investigate in‐depth the long‐term efficacy and safety of such novel approach.

In conclusion, this study demonstrated that the novel dose adjustment schedule that used an initial dose of 10,000 SQ was equally effective and is a safe alternative to the conventional schedule for delayed SCIT of more than 16 weeks for AR, with the advantage of saving time and reduced dropout rate. Besides, the interrupted SCIT do not exert a significant influence on overall clinical efficacy compared to a continuous schedule.

## CONFLICT OF INTEREST

All authors declare no financial or commercial conflicts of interest.

## AUTHOR CONTRIBUTIONS

**Lin Xi:** Investigation (equal). **Chengshuo Wang:** Investigation (equal). **Yunbo Gao:** Methodology (equal). **Yuan Zhang:** Investigation (equal); Methodology (equal); Writing – original draft (equal). **Luo Zhang:** Conceptualization (equal); Funding acquisition (equal); Investigation (equal); Methodology (equal); Project administration (equal); Writing – original draft (equal).

## Supporting information

Table S1
