# Supplementary material for: Comparative study of novel dosing schedules for interrupted immunotherapy for allergic rhinitis
Source: Clin Transl Allergy. 2022 Apr 15;12(4):e12147. doi: 10.1002/clt2.12147 (PMC9012097; doi:10.1002/clt2.12147)
Supplement: Supplementary file 1 — Table S1 [file CLT2-12-e12147-s001.docx]

**TABLES**

**Table E1.** Demographic and clinical characteristics of study participants.

| **Treatment group** | **Novel dose adjustment group (N=32)** | **Conventional dose adjustment group (N=26)** | **Routine cluster SCIT group (N=34)** | ***P* value** |
| --- | --- | --- | --- | --- |
| Age, years | 28.5 (13.3–34.8) | 20.0 (7.0–32.3) | 24.5 (11.8–34.3) | 0.140 |
| Sex, male/female | 20/12 | 17/9 | 28/6 | 0.164 |
| SCIT duration, months | 18.0 (14.0–20.0) | 17.5 (13.0–20.0) | 18.0 (16.0–20.0) | 0.694 |
| Late injection time, weeks | 17.3±1.2 | 17.9±1.5 | - | 0.090 |
| *Der p* sIgE, kUA/L | 2.3 (0.9–8.3) | 5.1 (0.9–32.0) | 6.5 (2.5–15.1) | 0.147 |
| Combined with *Der f* allergy, No. (%) | 20 (90.9%) | 15 (88.2%) | 31 (100%) | 0.174 |
| Total IgE, kU/L | 182.5 (79.8–304.5) | 194.0 (61.5–589.0) | 211.0 (95.3–444.0) | 0.756 |
| CSMS | 2.0 (1.8–3.3) | 2.9 (2.0–3.5) | 2.5 (1.4–3.5) | 0.356 |
| TNSS | 5.0 (3.3–7.0) | 5.0 (4.0–7.0) | 5.0 (4.0–7.0) | 0.658 |
| MS | 1.0 (0–2.0) | 1.5 (1.0–2.0) | 1.5 (0–2.0) | 0.448 |

SCIT: subcutaneous immunotherapy; CSMS: Combined symptom and medication score; TNSS: total nasal symptom score; MS: medication score
